# Minimally Invasive Cardiac Surgery: Sternal Incision Size *vs.* Extracorporeal Circulation Time and Other Factors

**DOI:** 10.21470/1678-9741-2024-0090

**Published:** 2026-01-29

**Authors:** Mesut Engin, Mustafa Abanoz, Ahmet Kagan AS, Umut Serhat Sanrı

**Affiliations:** 1 Department of Cardiovascular Surgery, University of Health Sciences, Bursa Yuksek Ihtisas Training and Research Hospital, Yildirim, Bursa, Turkey; 2 Department of Cardiovascular Surgery, University of Health Sciences, Mehmet Akif Inan Training and Research Hospital, Sanliurfa, Turkey

Dear Editor,

We have read the article by Cammertoni et al.^[1]^, entitled “Outcomes of Minimally Invasive Aortic Valve Replacement in Obese Patients: A Propensity-Matched Study”, with great interest. First, we congratulate the authors for their good contribution to the literature. However, we would like to discuss some points about minimally invasive cardiac surgery.

As in all surgical fields, minimally invasive procedures are becoming more common in cardiovascular surgery. With developing technology, these initiatives will become more widespread. In the current study, the authors compared the clinical outcomes of patients who underwent surgical aortic valve replacement (AVR) through two different incisions (partial upper sternotomy *vs.* conventional full sternotomy). They found a significantly shorter need for intubation, less need for continuous positive airway pressure, and less intensive care stay in patients with minimal incisions^[1]^.

The current study is a retrospective analysis covering nine years^[1]^, and there may have been some changes in adult cardiac surgery during this period. In recent years, del Nido cardioplegia solution (dNCS) has begun to be used in adult cardiac surgery. In a study conducted on patients undergoing AVR, positive effects on perfusion times and postoperative blood product use were found in patients using dNCS compared to blood cardioplegia^[2]^. Did the authors use the same cardioplegia solution in all patients they included in the study during the nine years? Was dNCS used in patient groups? This may indirectly affect postoperative respiratory functions.

One of the important issues in AVR surgery is the selection of prosthetic valves. One of the important questions about the study by Cammertoni et al.^[1]^ is whether a prosthesis suitable for the body surface area was selected for all patients included in the study. Was there a need for aortic root enlargement? In addition, the type of prosthetic valve rather than the size of the prosthetic valve may affect early clinical results. In a study conducted by Algarni et al.^[3]^, it was shown that there may be early postoperative hemodynamic differences in different types of prosthetic valves.

More tissue damage occurs due to larger surgical incisions. In other words, there is more complement activation^[4]^. Therefore, increased inflammation may increase early complications. However, cardiopulmonary bypass (CPB) also causes complement activation. Long CPB durations also mean more inflammation^[5]^. As a result, the advantages of small incisions will become evident due to the decrease in CPB times in minimally invasive methods with technical developments.

In their study, the authors evaluated patients with severe chest deformities, ascending aorta calcifications, and previous chest irradiation as contraindications for mini-sternotomy^[1]^. How many of the patients who underwent AVR using the conventional method had these features? Thorax deformities can also affect postoperative respiratory functions^[6]^.

As a result, minimally invasive methods will be increasingly used with developing technology. However, it may be too early to say that it is superior to the conventional method in terms of postoperative results^[7]^. However, from our clinical experience, we can say that mini sternotomy AVR could be performed to obtain a better cosmetic result with the same surgical risk.
